# The Influence of Time and Storage Conditions on the Antioxidant Potential and Total Phenolic Content in Homemade Grape Vinegars

**DOI:** 10.3390/molecules26247616

**Published:** 2021-12-15

**Authors:** Justyna Antoniewicz, Joanna Kochman, Karolina Jakubczyk, Katarzyna Janda-Milczarek

**Affiliations:** Department of Human Nutrition and Metabolomics, Pomeranian Medical University in Szczecin, 24 Broniewskiego Street, 71-460 Szczecin, Poland; kaldunskajustyna@gmail.com (J.A.); kochmaan@gmail.com (J.K.); jakubczyk.kar@gmail.com (K.J.)

**Keywords:** natural fermentation, spontaneous fermentation, chaptalisation, flavonoids, polyphenols

## Abstract

Fermented foods have been an integral part of the cuisines of almost all cultures in the world. In recent years, they have gained ground again, mainly due to their potential health benefits. One such product is grape vinegar, which apart from characteristic taste, is also a source of compounds with antioxidant activity. The aim of the study was to determine the changes in the antioxidant potential and the content of polyphenols that occur during the storage of grape vinegar obtained by spontaneous fermentation. The research material consisted of vinegar made from different white grape varieties grown in Poland. For each variety, two variants were prepared: with and without the addition of sugar in the fermentation process. The antioxidant potential, polyphenol content, soluble solids content and pH were monitored both during the two-month fermentation process and the subsequent 6-months storage under various conditions. Storage conditions and time of the storage affected the antioxidant activity and polyphenol content. The content of these compounds was also influenced by the variety of grapes used as well as the method of vinegar preparation.

## 1. Introduction

Vinegar is a product of the conversion of ethanol to acetic acid by acetic acid bacteria (AAB). For centuries, vinegar has been widely used all around the world, notably as a food preservative and a condiment. It is an essential ingredient of fruit and vegetable pickles and features prominently in mayonnaise, mustard and dressing recipes [1]. Vinegar can be made from fruit and many other products containing sugar. The most common source materials include grapes, apples, malt, honey, potatoes, rice and other products with a high sugar content [2], as well as alcohols, including wine [3]. Among the vinegars made from grapes and grape products, one can distinguish balsamic vinegar, wine vinegar (white and red) and also sherry vinegar [4]. The production of wine vinegar is particularly popular in countries with a warmer climate, otherwise known for wine production. According to the Observatory of Economic Complexity (OEC), the leading vinegar producers for 2018 were Italy, Spain, France and Germany [5]. According to the data of the Council of the European Union, Poland belongs to zone A of viticulture in Europe [6]. This zone is characterised by lower temperatures, which makes the growing conditions more demanding [7]. However, there is a significant increase in wine production in European countries with cooler climates. According to the data of the National Center for Agricultural Support, in the years 2009–2020, there was an almost 35-fold increase in the volume of wine production in Poland. During these years, viticulture grew from 36 to over 560 hectares [8].

In countries with a cooler climate, the use of the chaptalisation process is allowed. Chaptalisation refers to the addition of sugar during fermentation. This procedure is used mainly in wine making to achieve a higher alcohol content by increasing the amount of fermentable substrate for the yeast [9,10]. This process is used when, as a result of bad weather, the grape fruit cannot reach the appropriate level of maturity and, consequently, the appropriate level of sugar. The addition of sugar is also observed in the home production of fruit vinegars.

Vinegar is made in a two-step process. First, fermentable sugars are converted to ethanol by the action of yeast. The reaction takes place in anaerobic conditions. Afterward, AAB converts ethanol into acetic acid in aerobic conditions [11]. AAB are strictly aerobic microorganisms, Gram-negative or Gram-variable, belong to the family *Acetobacteriaceae* and are ubiquitous in nature [12]. Nowadays, 19 bacterial genera are included in this category, and the main species responsible for vinegar production represent the genera *Acetobacter*, *Gluconacetobacter, Gluconobacter* and *Komagataeibacter*, given their high capacity to convert ethanol to acetic acid and the resistance to high concentrations of acetic acid in their environment [13]. The specific composition of AAB found in any given vinegar may vary, being strongly determined by the nature of the source material and the particulars of the production process [14].

Nowadays, the following three approaches are used in vinegar production on an industrial scale: surface culture fermentation methods, submerged fermentation and generator methods [3]. Vinegar can also be obtained through the spontaneous fermentation of alcohol or fruit, which is a centuries-old method of food preservation. The process requires an alcoholic substrate, acetic acid bacteria and access to oxygen. It is initiated by the microorganisms naturally found in fruit. This is the oldest method, which has been used for centuries, e.g., in wine making [15], today also known as “natural fermentation” [16]. The fermenting culture is located on the surface of the liquid and is in direct contact with air; therefore, the method is referred to as the static method of vinegar production [17]. Usually, the spontaneous fermentation process is allowed to continue for 60 days [18]. Compared to contemporary production methods, it is a time-consuming process, and the final product is inferior in quality to vinegars obtained using industrial methods [19]. The microorganisms involved in fermentation, through their metabolic processes, lower the carbohydrate content, increasing that of organic acids, ethanol, etc. This process, in turn, lowers the risk of the proliferation of undesirable microorganisms, while at the same time improving the shelf-life and safety of the fermented product. These metabolites also enhance the palatability and sensory quality of the finished product [20]. The microbiota involved in the fermentation process can exert both positive and negative effects on the quality features and safety of the final product. These microorganisms can modulate the content of biological and chemical compounds in the finished product, which may affect its safety for consumption [21], for example, through contamination with mycotoxins produced by certain fungi [22]. In addition, the microorganisms involved in fermentation, by releasing volatile organic compounds, impact the aroma and palatability [23]. They also influence the content of macro- and micronutrients, modifying their amounts, bioavailability and digestibility [24]. The qualitative and quantitative composition of the microbial communities inhabiting grapes is affected by a range of factors, such as precipitation, ambient temperature, degree of ripeness of the fruit, presence of physical damage, use of agricultural practices in the vineyard [25], but also grape variety, geographical location or vineyard age [26]. At the end of fermentation, fruit vinegar contains organic acids, mainly acetic acid [17], colouring matter, mineral salts and other fermentation products, such as esters, ketones and aldehydes, which impart distinctive flavour and aroma to vinegar [27,28], as well as a range of compounds with antioxidant properties. 

The antioxidants found in vinegars may originate from the source material (e.g., fruit) [29] and may also arise in the course of the fermentation process [30]. One of the most numerous antioxidant compounds are polyphenols. Their content enhances the antioxidant potential, but also affects the colour and astringency of the vinegar. The final quantitative and qualitative composition of vinegar also depends on the availability of oxygen during the fermentation process [31]. Research shows that an increased intake of food rich in polyphenols is associated with a lower incidence of certain chronic diseases and mortality related to cardiovascular diseases [32,33,34]. According to our current state of knowledge, vinegar has been shown to lower blood pressure, counteract the effects of diabetes and prevent the development of cardiovascular diseases [35,36] and fatty liver [37]. It is also a powerful antibiotic [38] and antioxidant [39]. Its health benefits are related to the content of nutrients and bioactive substances [1], which make it a functional food [40]. The main bioactive compounds in fruit vinegars include organic acids, fructooligosaccharides, minerals and vitamins [41]. It is believed that a particularly important role in the prevention of certain chronic diseases is played by the polyphenols found in grapes. It was reported that their consumption can be linked to a reduced risk of arterial hypertension and other cardiovascular diseases [42]. 

For many years, wine vinegar was regarded as a cheap by-product of wine production. Nowadays, however, they are increasingly appreciated [43]. There is a high demand for high-quality grape vinegars, rich in compounds with beneficial effects on human health. Unfortunately, to date, there have been few scientific reports analysing the effects of individual grape varieties [44,45], vinegar fermentation processes and storage conditions [46] on the content of bioactive compounds. There is also a lack of studies dedicated to vinegars obtained by spontaneous fermentation.

Therefore, the aim of this work was to examine the changes in antioxidant potential, including the total content of flavonoids and phenolic compounds, during the fermentation and storage of grape vinegars under different conditions. The idea behind the above research was to determine whether the selection of the grape variety grown in Poland, the way of conducting the fermentation process and further storage of the finished product has an impact on the properties of the finished vinegar. Thus far, there has been no research on the subject of homemade grape vinegars, especially from varieties grown in Poland.

## 2. Results and Discussion

### 2.1. Changes of the Analysed Parameters Occurring during the Fermentation Process

The changes in the antioxidant potential, Total Polyphenol Content and Total Flavonoid Content, as well as pH and soluble solids content in grape vinegars during the fermentation process are presented in Table 1.

The antioxidant potential of vinegar samples changed over time during fermentation. The vinegars made with added sugar showed a higher antioxidant potential after the second month of fermentation. In the case of vinegars made without added sugar, those made from Solaris and Johanniter grapes showed higher reducing power after the first month of fermentation, while in vinegar made from Souvignier gris, the antioxidant potential was higher after the second month of fermentation. The highest antioxidant potential was found in vinegar made from Solaris grape with added sugar at the end of two months of fermentation (72.46% inhibition of DPPH). 

The fermentation time affected the Total Polyphenol Content of the studied vinegars too. Phenolic compounds are the main substances with antioxidant properties found in plant products [47]. Particularly high levels of these compounds are found in grapes [48,49], which were the source product used to make the studied vinegars. The main representatives of phenolic compounds are phenolic acids and flavonoids [50]. Extending the fermentation process up to two months had a strong effect on increasing the total phenolic content in all the samples included in the analysis. The trend was observed both in samples containing added sugar and in those with no sugar added to the fermentation process. Phenolic compounds can often occur in a cell-wall bound, glycosylated or polymerised form, which significantly affects their bioavailability. Fermentation may lead to the liberation of phenolic compounds and their conversion into more active forms [51]. The highest TPC was noted in vinegar made from the Solaris grape with added sugar after 2 months of fermentation (681.73 ± 14.55 mg/L). 

A comparative analysis of the variants with and without added sugar demonstrated that after the first month of fermentation, the vinegars made with added sugar had a lower phenolic content than those made without added sugar. The differences were statistically significant (Solaris *p* = 0.040405, Johanniter *p* = 0.000036, Souvignier gris *p* = 0.000036) (Table 2). Referring to the antioxidant potential, it can be observed that the addition of sugar had a statistically significant effect on the results obtained for vinegar from the Solaris (*p* = 0.000037) and Johanniter (*p* = 0.002010) varieties after the second month of fermentation. In the case of the Souvignier gris variety, the addition of sugar had a statistically significant effect on the differences in the antioxidant potential, both after the first (*p* = 0.002010) and the second month of fermentation (*p* = 0.000035). After the second month of fermentation, the vinegar variants with added sugar obtained from Solaris and Souvignier gris grapes had a higher content of polyphenols than their counterparts produced without the addition of sugar. Only in the case of the Johanniter grape variety was a higher phenolic content observed in the variant with no added sugar. Statistically significant differences between the variants (with vs. without added sugar) after 2 months were only observed for the Solaris grape (*p* = 0.000035) (Table 2). 

The increase in the content of phenolic compounds, determined with the use of the Folin-Ciocalteu reagent, may also be related to the specificity of the method. It is based on a redox reaction, which can also take place between other reducing agents, e.g., sugars, carotenoids or amino acids from the tested samples, which may lead to overestimation of the obtained results. However, it is a common method used to determine total phenolic compounds in food samples [52].

The total flavonoid content in the studied vinegars was also correlated with fermentation time. Extending the fermentation time up to two months had a statistically significant effect on increasing the TFC of the studied vinegars, irrespective of the variant (with and without added sugar). The vinegar made from the Souvignier gris grape (variant with no added sugar) was the sole exception in that its flavonoid content was not significantly affected by extending the fermentation time. The vinegar from the Johanniter variety (with added sugar) showed the highest TFC after 2 months of fermentation (309.10 ± 15.02 mg/L) among the examined vinegar samples. The comparative analysis of the variants with and without added sugar only returned statistically significant differences in TFC for the vinegars made from the Johanniter grape after one month of fermentation (*p* = 0.000242) (Table 2). The rise in TFC could also have been influenced by the activity of yeast during alcoholic fermentation. Research indicates that *S. cerevisiae* secretes enzymes such as alcohol dehydrogenase, amylase, β-glucosydase, invertase and proteases during fermentation and may cause an increased secretion of phenolic compounds, for example, by disintegrating plant cell walls [53]. It should also be considered that the evaporation of the samples, and consequently, the product concentration may also have contributed to the increase in the levels of bioactive compounds [54]. The levels and composition of individual phenolic compounds are affected by the type of vinegar and production method [55].

The fermentation of plant materials with a variety of microorganisms, including lactic acid bacteria, filamentous fungi and yeast, can lead to an increase in phenolic compounds. This phenomenon is influenced by a number of metabolic pathways, such as glycosylation, deglycosylation, methylation, glucuronidation and sulfate conjugation [56]. The increase in the content of phenolic compounds was also observed by Liu et al., where the fermentation process carried out with the participation of *L. plantarum* and *S. cerevisiae* significantly influenced the content of flavonoids [57].

However, it should be borne in mind that natural fermentation is characterised by a large, often uncharacterised diversity of microflora; therefore, the metabolism processes taking place in the products obtained with this method are also unknown. Research on the spontaneous fermentation of red grape varieties from Poland showed that the succession of different yeast groups changed along with the process. At the initial stage of fermentation, the development of *Hanseiaspora uvarum* and *Candida railenensis* was observed, which were then replaced by *Saccharomyces cerevisiae* [58]. The occurrence of these strains is characteristic of the colder climatic zone A of grapevine cultivation. The pH of the studied samples was analysed as well. Surprisingly, extending the fermentation time up to two months was associated with an increase in pH in the vinegars made without added sugar compared to the results obtained after the first month of fermentation. This increase is related to the increasing content of organic acids, which are products of fermentation [58]. After the two-month period of fermentation, the pH of the analysed vinegar samples ranged from 2.96 ± 0.02 to 3.52 ± 0.04. Similar results were obtained by Bozemir et al. [59], where the pH of grape vinegars amounted to between 2.84 ± 0.01 and 2.95 ± 0.02. An extended fermentation time also altered the pH in the vinegars made with added sugar, but the changes were not statistically significant (3.02 ± 0.06 after the first month of fermentation vs. 3.05 ± 0.03 after the second month of fermentation for the Solaris grape, 3.25 ± 0.14 vs. 3.23 ± 0.09 for the Johanniter grape and 2.96± 0.02 vs. 2.96 ± 0.01 for the Souvignier gris grape). A statistically significant difference in pH between the first and second month of fermentation was observed only for the Solaris grape (variant with sugar) (*p* = 0.009375). The comparison of pH values between variants after the first and second month of fermentation showed that the vinegars made with added sugar had a lower pH than their unsweetened counterparts (respectively, after the first and second month: Solaris *p* = 0.000693, *p* = 0.000089; Johanniter *p* = 0.000032, *p* = 0.000032; Souvignier gris *p* = 0.004157, *p* = 0.000029). 

The use of the chaptalisation process had a significant impact on the content of soluble solids. These compounds include among others glucose, fructose, sucrose, as well as organic acids and free amino acids [60]. The levels of these compounds may be additionally affected by the characteristics of the source material and bacterial cultures involved in fermentation [18]. The content of soluble solids in all the samples under analysis declined with the time of fermentation. At the end of the first month of fermentation, the vinegar variants with added sugar made from Solaris and Johanniter grapes had higher levels of soluble solids compared to their counterparts without added sugar (respectively, *p* = 0.000029, *p* = 0.008829). The higher the microbial fermentation activity was, the lower the sugar content was. In turn, the pH of vinegars depends on the source material used in their production [18].The difference between the variants of the Souvignier gris grape was not statistically significant (4.5 ± 0.27 °Brix for the variant without added sugar vs. 4.5 ± 0.34 °Brix for the variant with sugar). On the other hand, statistically significant differences in the content of soluble solids after the second month of fermentation between variants (with chaptalisation vs. without added sugar) were observed in the vinegars made from Solaris and Johanniter grapes (respectively, *p* = 0.000089 and *p* = 0.000196). The statistical analysis uncovered a range of significant differences in the analysed parameters between the vinegars made from different grape varieties. The results are presented in Table S1 (Supplementary Materials). It shows that the variety of grapes used in most cases had a significant impact on the differences in the values of the analysed parameters during the fermentation process of both vinegar variants.

During the fermentation process, the influence of the chaptalisation process on obtaining differences was clearly visible in the content of polyphenols, flavonoids and total antioxidant capacity. It was especially apparent that the total phenolic content was higher after one month of fermentation in the samples without added sugar compared to those with added sugar. This phenomenon may be related to the higher osmotic pressure in the samples containing added sugar. On the other hand, increased osmotic pressure may have a restrictive effect on the development of certain yeast strains [61], which means reactions may have occurred at a slower rate, and consequently, the bioavailability of phenolic compounds would be lower than in the samples without added sugar. 

### 2.2. Changes of Analysed Parameters during Storage under Refrigerated Conditions

After the fermentation process was completed, the analysed vinegar samples were stored in a refrigerator and at room temperature. Existing research on the influence of storage on the properties of vinegars is mainly limited to the maturation processes [62], including those carried out in wooden barrels [63]. In some cases, wooden chips can also be added to vinegars [17]. The direct contact of the product with the wooden barrel and the microdiffusion of oxygen through wood pores affects factors such as colour, aroma, as well as the amount and characteristics of phenolic compounds [64]. However, there is no sufficient studies analysing changes during vinegar ripening in glass bottles [44]. 

**Figure 1:**
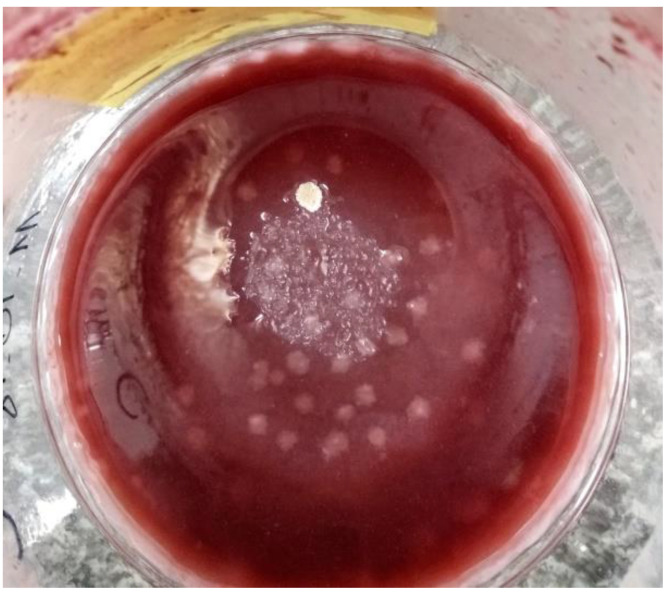
Visible mould on the surface of the vinegar.

In our study, we analysed the antioxidant potential, TPC, TFC, as well as pH and soluble solids content in the vinegar samples during the 6-month storage process in the fridge (Table 3). Refrigeration is one of the methods of food preservation, and it can also be used with vinegar. Lower temperatures slow the pace of oxidation reactions but cannot eliminate them altogether. Oxidation processes, in turn, lead to further chemical and enzymatic reactions, which can affect the quality of the stored product [65]. In our study, spoilage occurred in vinegars made from Souvignier gris grape, with mould forming on the surface. In the samples made without added sugar, mould appeared after 6 months of storage, and in the variant with added sugar, adverse changes had already appeared in the third month of storage (Figure 1). For this reason, these samples were not included in further analyses. Moulds occurring on the surface of vinegars are mainly representatives of the genus *Aspergillus*. They show high metabolic activity, producing, among others, mycotoxins, chiefly aflatoxins and ochratoxins [66], which are harmful to humans and animals, with hepatotoxic, nephrotoxic and carcinogenic effects [67]. It has also been reported that mycotoxins were found in other grape products, including wine [68,69]. According to Schindhelm et al. [70], moulds are the main type of microorganisms contaminating balsamic vinegars and cider vinegars.

In the majority of the analysed samples, the highest antioxidant potential was observed after one month of storage (Table 3). An extended storage time was associated with a gradual decline in the antioxidant potential of the samples. The vinegar made from the Johanniter grape (variant with no added sugar) was an exception, with the highest percentage inhibition of DPPH noted after 3 months of storage. However, in this case too, further storage led to a decline in the antioxidant potential. On the other hand, the antioxidant potential of vinegar made from the Souvignier gris grape (with added sugar) measured at baseline and after 1 month of storage was the same. Out of all the vinegar samples stored in the refrigerator, the highest antioxidant potential (82.01% inhibition of DPPH) was observed in the vinegar made from the Johanniter grape (variant with added sugar) after 1 month of storage. Studies showed that the total phenolic content is directly proportional to the ripening time of the vinegar. Xu et al. [71] observed that the antioxidant potential of vinegar is significantly affected by the storage process.

The TPC and TFC changed dynamically over the course of storage. The levels of both parameters changed correspondingly. For both variants of vinegar made from the Solaris grape, the highest level of compounds under analysis was observed after the third month of storage. Studies showed that the total phenolic content is correlated with antioxidant activity [72,73]. We observed that irrespective of the storage conditions (refrigerated storage or room temperature), the highest antioxidant potential, TPC and TFC were noted in the vinegars made from the Johanniter grape. This is a clear indication that grape variety has an effect on the content of antioxidants. The TPC in the samples in our study was as high as 1027.48 mg/L. Significantly lower results, in the range of 306 ± 4–867 ± 7 mg/L, were obtained by Davalos et al. [72]. The observations by Andlauer et al. were lower still [74], with the TPC of white wine vinegars falling in the range 205–509 mg/L. This was similar to the TPC of white wines, amounting to 211–592 mg/L. Likewise, Lugasi et al. [75] noted the TPC in white wines amounting to 250–567 mg/L. However, the lower levels of phenolic compounds and antioxidant activity in white wines compared to the studied vinegars may be attributable to the different production method. White wine is made by using mainly grape juice—grape skins are removed and play no role in fermentation [76]. At the same time, it is known that the highest concentrations of phenolic compounds are found in grape skins. In our study, whole fruits were used in the fermentation process, and hence bioactive compounds could pass freely into the final product. More and more often, it is emphasised that contact with grape skins during the fermentation process increases the content of bioactive compounds in the final product [77]. On the other hand, acetic fermentation may lower the content of phenolic compounds or lead to the production of new compounds with a lower antioxidant potential than those found in the wine used to make vinegar [75]. 

With respect to the vinegars made from the Johanniter grape, the highest levels of these parameters were noted after 6 months of storage for the variant without added sugar and 3 months of storage for the variant with added sugar. The highest levels of TFC and TPC in the unsweetened variant of the vinegar made from the Souvignier gris grape were also determined after 3 months of storage. On the other hand, the variant with added sugar of the same grape variety contained the highest amounts of both parameters immediately upon the completion of the fermentation process. The storage process led to the decline of both the TFC and TPC, and subsequently, mould growth.

All of the samples included in the analysis showed a marked initial decline in the TPC and TFC after the first month of storage, compared to the initial measurements. Afterward, during further storage, the parameters increased. 

Over the course of storage, changes were also observed in the pH of vinegars. In the majority of cases, the extension of the storage time had a statistically significant impact on the obtained results. Changes were also observed in the content of soluble solids in the analysed samples. However, depending on the sample, maximum contents were noted at different time points. A comparison of the significance of the differences of the antioxidant potential (DPPH), Total Polyphenol Content (TPC), Total Flavonoid Content (TFC), pH and soluble solids of the samples prepared from different grape varieties during the storage process under refrigerated conditions were presented in Table S2. Statistically significant differences were observed between vinegars prepared from different varieties of white grapes grown in Poland, when stored in the refrigerator. These differences concerned both the variant of preparing vinegars without added sugar (variant A) and with its addition (variant B).

A comparative analysis was carried out to examine the effects of chaptalisation on the content of the parameters under analysis in the course of storage. The statistically significant differences in the analysed parameters between the variants (with vs. without added sugar) are presented in Table 4.

### 2.3. Changes of Analysed Parameters during Storage at Room Temperature

After the completion of the fermentation process, changes were also observed in the antioxidant potential, TPC, TFC, soluble solids and pH in the samples stored at room temperature. The results are presented in Table 5. However, in this case, the experiment was concluded at the end of the third month of storage. By then, mould developed in all the vinegar samples, and consequently further determinations were not carried out. While vinegar is regarded as a long-lasting product that can be stored over an extended period of time, storage can still change its physicochemical and sensory characteristics, due to processes such as oxidation and ageing [44]. Exposure to oxygen may also contribute to the development of pathological microorganisms and enhance oxidation. Along with the time of storage, the pH of vinegar samples increased up to 7.52 ± 0.01. Dissimilar results were obtained by Kang et al. [60], who found that in the majority of cases the pH of grape vinegars decreased during the storage period. The increasing pH values observed in our study may be attributable to the enhanced metabolic activity of other microorganisms found in vinegar. The growth of yeasts and moulds is also possible in an acidic environment (pH value 4.0 to 4.5 for yeast, 2.0–8.5 for mould) [19]. The moulds present in the environment take part in the alcoholic fermentation of vinegar. They also play an important role at a later stage, because of their capacity to produce secondary metabolites, including compounds with antibiotic effects [78]. Given these observations, it is evident that storing vinegars at room temperature was not beneficial for their safety and quality. 

With respect to both variants of vinegar made from the Johanniter grape and the unsweetened variant made from the Solaris grape, the highest antioxidant potential was noted at baseline, that is, immediately upon the completion of the fermentation process. Over the course of storage, the antioxidant potential gradually declined. For the remaining samples, the highest values of antioxidant potential were determined after one month of storage. The highest antioxidant potential (74.15% inhibition of DPPH) out of the vinegars stored at room temperature was noted for the Johanniter vinegar (variant with added sugar) at baseline, that is, immediately upon the completion of the fermentation process. 

The changes in the TPC and TFC in the respective samples over the course of storage ran parallel. For both variants of vinegars made from Solaris and Souvignier gris grapes, the highest values of both parameters were noted at baseline. In turn, both variants of vinegar made from the Johanniter grape demonstrated the highest values of the above parameters after 3 months of storage. 

With respect to pH levels, over the course of storage, the pH increased in all the samples, reaching peak values after 3 months of storage. The opposite was observed in the analysis of soluble solids content. Their maximum values were determined immediately upon the completion of the fermentation process, decreasing with the passage of storage time. A similar observation was also made by Kang et al. [60]. 

Only in the case of the Johanniter vinegar made with added sugar was the highest content of soluble solids reached after one month of storage. Further storage was associated with a decrease in this parameter. The effects of chaptalisation on the changes in the analysed parameters during storage are presented in Table 6. The statistical analysis clearly shows that the addition of sugar in most cases had a statistically significant effect on the obtained values of the analysed parameters during the storage of vinegar samples at room temperature. Additionally, in Table S3, a comparison of the significance of the differences of the antioxidant potential (DPPH), Total Polyphenol Content (TPC), Total Flavonoid Content (TFC), pH and soluble solids of the analysed samples prepared from different grape varieties during the storage process at room temperature is shown. There are statistically significant differences between the vinegars prepared from different grape varieties during storage at room temperature. These differences occurred in both variants of vinegar preparation (without sugar and with its addition).

Vinegar storage in glass bottles was investigated by Davies et al. [44]. No significant changes in the content of phenolic compounds were observed during storage, but the antioxidant potential was reduced. According to the literature data, storage in oak bar-rels has a positive effect on the content of phenolic compounds. Furthermore, Llabe Pino [79] observed reduced levels of hydroxybenzoic and hydroxycinnamic acids and their derivatives in Chardonnay, Cabernet Sauvignon, Moscatel and apple vinegars after one year of storage in glass bottles at 15 °C. Additional observations included the formation of new compounds, related, e.g., to enzymatic browning reactions. In view of the above, there appears to be good reason to determine the detailed profile of these compounds in order to explore these changes in detail, which is planned as the next step of our re-search.

## 3. Materials and Methods

### 3.1. Grape Vinegars Preparation

The vinegars used in this study were made from the fruit of white wine grape varieties (*Vitis vinifera* L.) obtained from a vineyard in the Western Pomerania region of Poland (53°15′35″ N 14°43′24″ E) in September 2018. The study used Solaris, Johanniter and Souvignier gris white-coloured grape varieties. Grapes were collected during the full ripening stage. Bunches of mature grapes were collected from several grape vines within an area of the vineyard. The stems and leaves were removed from the grapes. A total of 250 g of randomly selected berries were placed in sterile glass jars and pressed until juice covered the fruits. For each variety, vinegars were prepared according to two different procedures. In variant A, only crushed fruit and distilled water were used at a 1:1 mass ratio (250 g of berries + 250 mL of distilled water). In variant B, additionally, the chaptalisation process was used: a solution of distilled water and table sugar (70 g sugar per 1 L of water) was added to the fruit (also 1:1 mass ratio). Vinegar was produced by spontaneous fermentation under constant thermal conditions (24 °C) over a period of two months, carried out by the natural flora inhabiting the fruit. Jars were covered with sterile gauze to ensure air access. The vinegars were also mixed twice a day. The process was carried out for 60 days. The analyses were carried out after 1 and 2 months of fermentation. Both variants of the fermentation process were performed in triplicate.

**Figure 2:**
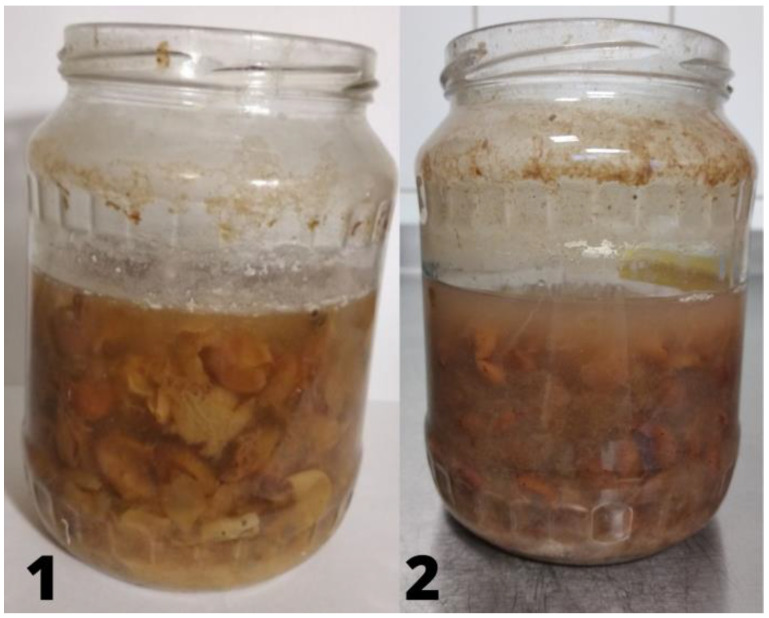
Grape vinegar samples after 1st and 2nd month of fermentation.

Figure 2 shows the changes taking place in the vinegar samples tested after the first (1) and second month (2) of fermentation.

### 3.2. Storage 

**Figure 3:**
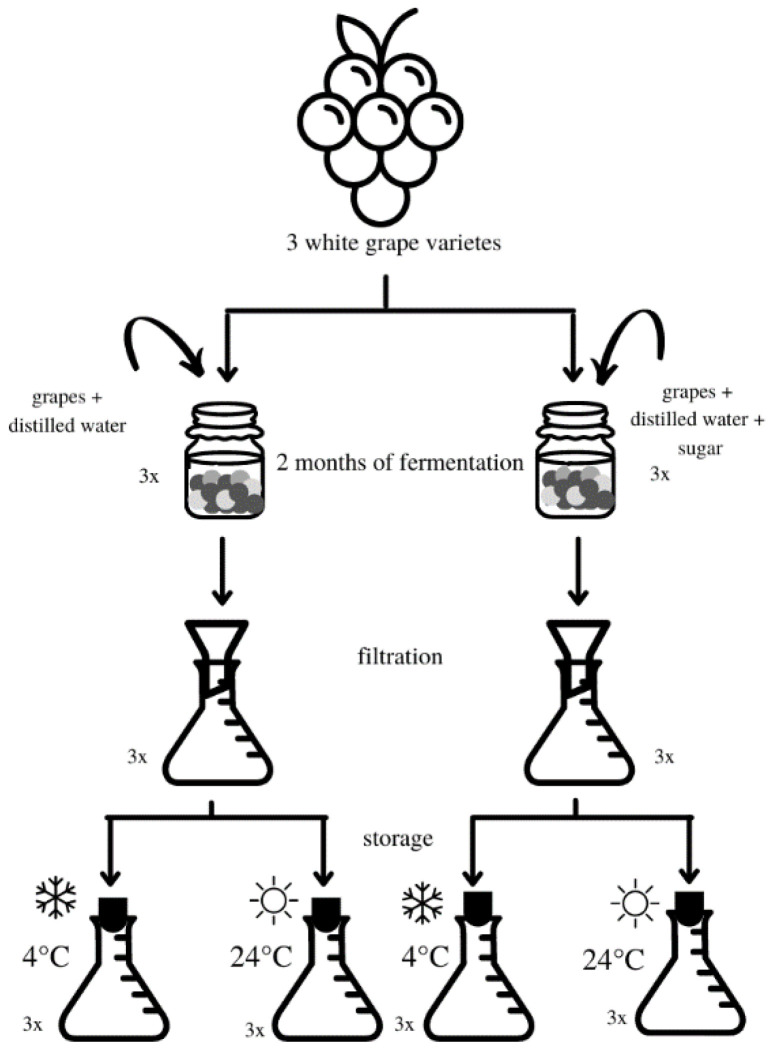
Flow chart of vinegar sample preparation and storage process.

After two months of fermentation, fruit residues were removed, and the vinegars were filtered. Each of the variants were then subjected to storage in a dark, tightly closed glass vessel under the following two different sets of conditions: in a dark place at room temperature (24 °C) and under refrigerated conditions (4 °C). The scheme of samples received during preparation and subsequent storage is presented in Figure 3. Samples were stored for 6 months. Analyses were carried out at the following time points: the first, third and sixth month of the storage. All vinegar variants used in the analyses were prepared in triplicate. 

### 3.3. Determination of Total Antioxidant Activity

The antioxidant activity of analysed samples was measured using spectrophotometry (Agilent 8453 UV-visible spectrophotometer) with a DPPH (2,2-diphenyl-1-picrylhydrazyl, Sigma Aldrich, Darmstadt, Germany) synthetic radical. The antioxidant activity of samples was measured according to Brand-Williams et al. and Pekkarinen et al. [80,81]. The spectral absorbance was measured at 518 nm. All assays were performed in triplicate. Antioxidant potential of tested samples has been expressed by the percent of DPPH inhibition, using the following formula:

(1) $$\%\;of\;inhibition=\frac{A0-As}{A0}\times 100$$

where

A0—absorbance of DPPH solution at 518 nm without tested sample;

As—absorbance of DPPH solution at 518 nm with tested sample.

### 3.4. Determination of the Total Phenolic Content (TPC)

The polyphenol content of the analysed samples was assessed using the Folin–Ciocalteu reagent [82]. The absorbance was measured at 765 nm (8453UV, Agilent Technologies, Santa Clara, CA, USA). Polyphenol content was calculated from the calibration curve plotted using gallic acid as the reference standard. The results are shown as mg of gallic acid in 1 L of liquid (mg GAE/1 L). All assays were performed in triplicate.

The polyphenol content of the analysed samples was assessed using the Folin–Ciocalteu reagent [82]. The absorbance was measured at 765 nm (8453UV, Agilent Technologies, Santa Clara, CA, USA). Polyphenol content was calculated from the calibration curve plotted using gallic acid as the reference standard. The results are shown as mg of gallic acid in 1 L of liquid (mg GAE/1 L). All assays were performed in triplicate.

### 3.5. Determination of the Total Flavonoid Content (TFC)

Determination of the Total Flavonoids Content was performed according to the Pękal and Pyrzynska and Hu methods [83,84]. Different concentrations of flavonoids were used in the plotting of the standard calibration curve. The content of flavonoids was determined from the calibration curve using the rutin equivalent as the reference standard (0–120 mg/L of rutin equivalent). The absorbance was measured at 510 nm (8453UV, Agilent Technologies, Santa Clara, CA, USA). The results are shown as mg of rutin equivalent in 1 L of liquid (mg RE/1 L). All assays were performed in triplicate. 

### 3.6. Determination of pH

The pH of the analysed samples was determined using a pH meter (Schott Instruments; SI Analytics Mainz, Germany). All assays were performed in triplicate.

### 3.7. Determination of Soluble Solids

The total amount of soluble solids was measured using a laboratory refractometer RL3 (Polish Optical Works, Warsaw, Poland). Results were expressed using a Brix scale. All assays were performed in triplicate.

### 3.8. Statistical Analysis

In all the experiments, three samples were analysed, and all the assays were carried out in triplicate; therefore, the results of 9 replicates for each trial were used for statistical analysis. The statistical analysis was performed using Stat Soft Statistica 13.0 and Microsoft Excel 2017. The results are expressed as mean values and standard deviation (SD). Distributions of values for each parameter were analysed using the Shapiro–Wilk test. To assess the differences between examined parameters, the Kruskal–Wallis test was used. Differences were considered significant at *p* ≤ 0.05.

## 4. Conclusions

The antioxidant activity and content of the bioactive compounds in vinegars made by spontaneous fermentation depended on the grape variety, as well as the production method and fermentation time, but also the storage time and conditions of the finished product. With some grape varieties, chaptalisation was associated with a higher content of antioxidants. Moreover, storage conditions affected the vinegar quality and safety. The selection of the grape variety also had a statistically significant impact on the content of the analysed compounds, both during the vinegar fermentation process and their subsequent storage under various conditions. This study demonstrates that an extended storage time of vinegar, both at room temperature and under refrigerated conditions, led to a gradual decline in antioxidant activity. Given the above findings, it is recommended that vinegars made by spontaneous fermentation should be fermented for two months, and afterwards kept in the refrigerator and used as soon as possible.

## Figures and Tables

**Table 1:** Changes in antioxidant potential, Total Polyphenol Content (TPC), Total Flavonoid Content (TFC), pH and content of soluble solids in vinegars during fermentation. The results are presented as mean ± standard deviation of 9 replications.

Grape Variety	Variant	Time Point (Month)	DPPH (%)	TPC (mg GAE/L)	TFC (mg RE/L)	pH	Soluble Solids (°Brix)
Solaris	A	1	70.13 ± 6.14	299.39 ± 13.16	214.16 ± 3.08	3.13 ± 0.15	4.35 ± 0.21
		2	66.31 ± 4.13	326.06 ± 5.46	303.32 ± 29.96	3.21 ± 0.15	3.50 ± 0.71
	B	1	68.52 ± 2.56	271.51 ± 35.04	229.25 ± 17.33	3.02 ± 0.06	5.60 ± 0.14
		2	72.46 ± 0.86	681.73 ± 14.55	298.98± 18.22	3.05 ± 0.03	5.00 ± 1.41
Johanniter	A	1	67.42 ± 5.35	280.97 ± 17.35	211.83 ±17.33	3.43 ± 0.04	3.05 ± 0.07
		2	55.39 ± 10.79	334.58 ± 4.68	288.79± 22.84	3.52 ± 0.04	3.00 ± 0.00
	B	1	66.53 ± 9.99	252.90 ± 3.60	164.16 ± 10.72	3.25 ± 0.14	3.30 ± 0.42
		2	70.98 ± 9.88	331.12 ± 8.80	309.10 ± 15.02	3.23 ± 0.09	2.00 ± 1.41
Souvignier gris	A	1	41.96 ± 4.85	275.86 ± 4.73	165.58 ± 13.59	3.00 ± 0.07	4.50 ± 0.27
		2	44.65 ± 0.17	308.07 ± 14.44	225.92 ± 16.58	3.18 ± 0.01	3.50 ± 0.00
	B	1	49.58 ± 3.07	231.54 ± 3.60	170.08 ± 9.30	2.96 ± 0.02	4.50 ± 0.34
		2	50.92 ± 0.50	312.58 ± 6.71	235.56 ± 179.78	2.96 ± 0.01	3.75 ± 0.35

Variant A—vinegars made only from crushed fruit and distilled water; variant B—vinegars prepared with the sugar addition (a solution of distilled water and table sugar was added to the crushed fruit).

**Table 2:** The *p*-values of the Kruskal–Wallis test for samples made with sugar (variant B) vs. without added sugar (variant A) during fermentation process.

Grape Variety	Fermentation Time [Month]	DPPH	TPC	TFC	pH	Soluble Solids
Solaris	1	ns.	0.040405	ns.	0.000693	0.000029
	2	0.000037	0.000035	ns.	0.000089	0.000089
Johanniter	1	ns.	0.000036	0.000242	0.000032	0.008829
	2	0.002010	ns.	ns.	0.000032	0.000196
Souvignier gris	1	0.002010	0.000036	ns.	0.004157	ns.
	2	0.000035	ns.	ns.	0.000029	0.003307

Results with *p* ≤ 0.05 were regarded as statistically significant. ns.—not significant.

**Table 3:** DPPH (Antioxidant potential), TPC (Total Phenolic Content), TFC (Total Flavonoid Content), pH and soluble solids content in vinegar samples during storage under refrigerated conditions (4 °C). The results are presented as mean ± standard deviation of 9 replications.

Grape Variety	Variant	Time Point (Month)	DPPH (%)	TPC (mg GAE/L)	TFC (mg RE/L)	pH	Soluble Solids (°Brix)
Solaris	A	0	68.78 ± 0.86	326.06 ± 5.46	303.32 ± 29.96	3.2 ± 0.151	3.50 ± 0.00
		1	72.78 ± 0.92	145.60 ± 0.48	89.33 ± 9.83	3.32 ± 0.01	3.75 ± 0.10
		3	61.48 ± 0.44	754.02 ± 22.46	343.09 ± 29.70	3.11 ± 0.04	3.75 ± 0.15
		6	57.33 ± 0.22	664.45 ± 5.49	334.51 ± 16.97	3.53 ± 0.01	3.00 ± 0.10
	B	0	69.43 ±0.49	681.73 ± 14.55	298.98 ± 18.22	3.05 0.03	5.00 ± 0.00
		1	70.28 ± 0.16	147.36 ± 0.10	77.52 ± 2.69	3.26 ± 0.01	4.25 ± 0.00
		3	64.41 ± 0.29	816.38 ± 8.91	540.88 ± 43.27	3.01 ± 0.01	4.50 ± 0.10
		6	53.08 ± 7.94	727.10 ± 5.53	300.74 ± 4.38	3.34 ± 0.00	3.75 ± 0.15
Johanniter	A	0	70.18 ± 7.38	334.58 ± 4.68	288.79 ± 22.84	3.52 ± 0.04	3.00 ± 0.10
		1	41.72 ± 0.17	141.70 ± 0.04	78.38 ± 8.62	3.28 ± 0.01	2.75 ± 0.00
		3	75.37 ± 0.20	753.76 ± 0.68	375.96 ± 14.21	3.52 ± 0.01	2.75 ± 0.15
		6	70.13 ± 3.14	1027.48 ± 0.92	535.41 ± 58.15	3.52 ± 0.01	2.00 ± 0.00
	B	0	70.08 ± 2.88	331.12 ± 2.88	309.10 ± 15.02	3.23 ± 0.09	2.00 ± 0.20
		1	82.01 ± 0.06	151.72 ± 0.41	114.79 ± 24.99	3.29 ± 0.04	3.50 ± 0.00
		3	78.38 ± 4.25	972.49 ± 2.02	637.48 ± 51.00	3.52 ± 0.01	3.25 ± 0.00
		6	81.83 ± 4.01	322.50 ± 4.18	271.28 ± 2.10	3.50 ± 0.00	1.75 ± 0.10
Souvignier gris	A	0	34.24 ± 0.14	308.07 ± 14.44	225.92 ± 16.58	3.18 ± 0.01	3.50 ± 0.00
		1	69.37 ± 0.64	145.19 ± 0.08	64.24 ± 7.07	3.26 ± 0.00	2.75 ± 0.10
		3	55.33 ± 2.99	390.77 ± 43.62	245.38 ± 19.63	3.31 ± 0.01	2.75 ± 0.00
		6	X	X	X	X	X
	B	0	31.92 ± 0.24	312.58 ± 6.71	235.56 ± 179.78	2.96 ± 0.01	3.75 ± 0.00
		1	31.92 ± 0.24	149.45 ± 0.04	104.57 ± 1.20	3.26 ± 0.01	4.25 ± 0.20
		3	X	X	X	X	X
		6	X	X	X	X	X

X indicates the samples on which the mould appeared. Variant A—vinegars made only from crushed fruit and distilled water; variant B—vinegars prepared with the sugar addition (a solution of distilled water and table sugar was added to the crushed fruit).

**Table 4:** The *p*-values of the Kruskal–Wallis test for samples made with (variant B) vs. without added sugar (variant A) during storage under refrigerated conditions.

Grape Variety	Time Point (Month)	DPPH	TPC	TFC	pH	Soluble Solids
Solaris	0	0.000037	0.000037	ns.	0.00011	0.00011
	1	0.008114	0.008114	0.008114	0.030348	0.030384
	3	0.005075	0.005075	0.005075	0.043309	0.030384
	6	ns.	0.005075	0.005075	0.030384	0.030384
Johanniter	0	0.001883	ns.	ns.	0.000055	0.000506
	1	0.008114	0.008114	0.008114	ns.	ns.
	3	ns.	0.005075	0.005075	0.030384	0.030384
	6	0.005075	0.005075	0.005075	0.030384	0.030384
Souvignier gris	0	0.000087	ns.	ns.	0.000248	0.008844
	1	0.005075	0.005075	0.005075	ns.	0.030374

Results with *p* ≤ 0.05 were regarded as statistically significant. ns.—not significant.

**Table 5:** DPPH (Antioxidant potential), TPC (Total Phenolic Content), TFC (Total Flavonoid Content), pH and soluble solids content in the analysed vinegar samples during storage at room temperature. The results are presented as mean ± standard deviation of 9 replications.

Grape Variety	Variant	Time Point (Month)	DPPH (%)	TPC (mg GAE/L)	TFC (mg RE/L)	pH	Soluble Solids (°Brix)
Solaris	A	0	68.19 ± 1.26	326.06 ± 5.46	303.32 ± 29.96	3.21 ± 0.15	3.50 ± 0.70
		1	66.66 ± 0.84	116.50 ± 0.08	86.41 ± 6.91	3.25 ± 0.00	2.75 ± 0.10
		3	57.51 ± 6.03	197.05 ± 0.43	92.05 ± 7.36	7.09 ± 0.01	2.00 ± 0.05
	B	0	68.87 ± 0.83	681.73 ± 14.55	298.98 ± 18.22	3.05 ± 0.03	5.00 ± 1.40
		1	71.35 ± 2.65	141.64 ± 0.40	95.82 ± 5.10	3.26 ± 0.01	3.75 ± 0.75
		3	65.70 ± 0.25	295.53 ± 7.35	136.15 ± 12.40	7.52 ± 0.01	2.00 ± 0.15
Johanniter	A	0	66.59 ± 0.92	334.58 ± 4.68	288.79 ± 22.84	3.52 ± 0.04	3.00 ± 0.00
		1	53.61 ± 3.32	149.74 ± 0.27	98.25 ± 7.86	3.25 ± 0.00	2.50 ± 1.10
		3	44.62 ± 0.72	505.47 ± 19.96	426.65 ± 34.56	4.45 ± 0.01	2.25 ± 0.20
	B	0	74.15 ± 6.62	331.12 ± 8.80	309.10 ± 15.02	3.23 ± 0.09	2.00 ± 1.41
		1	73.45 ± 0.11	148.25 ± 0.22	94.57 ± 7.03	3.26 ± 0.00	2.75 ± 0.45
		3	60.39 ± 1.35	822.97 ± 0.12	514.55 ± 27.79	4.03 ± 0.01	2.25 ± 1.00
Souvignier gris	A	0	67.66 ± 3.23	308.07 ± 14.44	134.19 ± 16.58	3.18 ± 0.01	3.50 ± 0.00
		1	69.25 ± 1.00	144.79 ± 0.09	66.61 ± 5.33	3.27 ± 0.00	2.50 ± 0.55
		3	60.25 ± 0.36	600.63 ± 0.55	225.92 ± 5.24	3.58 ± 0.00	2.50 ± 0.25
	B	0	34.22 ± 0.16	312.58 ± 6.71	235.56 ± 17.78	2.96 ± 0.01	3.7 ± 0.35
		1	66.95 ± 3.22	139.73 ± 0.51	62.86 ± 4.60	3.50 ± 0.01	2.75 ± 1.05
		3	61.92 ± 0.42	286.66 ± 5.87	166.00 ± 3.48	3.54 ± 0.01	2.75 ± 0.95

Variant A—vinegars made only from crushed fruit and distilled water; variant B—vinegars prepared with the sugar addition (a solution of distilled water and table sugar was added to the crushed fruit).

**Table 6:** The *p*-values of the Kruskal–Wallis test for samples made with (variant B) vs. without added sugar (variant A) during storage at a room temperature.

Grape Variety	Time Point (Month)	DPPH	TPC	TFC	pH	Soluble Solids
Solaris	0	0.000037	0.000037	ns.	0.000110	0.000110
	1	0.005075	0.005075	0.005075	ns.	0.030384
	3	0.005075	0.005075	0.005075	0.03084	ns.
Johanniter	0	0.001883	ns.	ns.	0.000055	0.000506
	1	0.008114	0.008114	0.008114	ns.	ns.
	3	0.005075	0.005075	0.005075	0.030384	ns.
Souvignier gris	0	0.000087	ns.	ns.	0.000248	0.012165
	1	ns.	0.008114	0.008114	ns.	0.030384
	3	0.005075	0.005075	0.005075	0.030384	ns.

Results with *p* ≤ 0.05 were regarded as statistically significant. ns.—not significant.

## Data Availability

The data presented in this study are available in this article.

## Supplementary Materials

The following are available online. Table S1: Comparison of the significance of differences of the antioxidant potential (DPPH), Total Polyphenol Content (TPC), Total Flavonoid Content (TFC), pH and soluble solids of the analysed samples during the fermentation process (after the first and second month of fermentation); Table S2: Comparison of the significance of the differences of the antioxidant potential (DPPH), Total Polyphenol Content (TPC), Total Flavonoid Content (TFC), pH and soluble solids of the analysed samples during the storage process under refrigerated conditions; Table S3: Comparison of the significance of the differences of the antioxidant potential (DPPH), Total Polyphenol Content (TPC), Total Flavonoid Content (TFC), pH and soluble solids of the analysed samples during the storage process at room temperature.
