# Supplementary material for: The Influence of Time and Storage Conditions on the Antioxidant Potential and Total Phenolic Content in Homemade Grape Vinegars
Source: Molecules. 2021 Dec 15;26(24):7616. doi: 10.3390/molecules26247616 (PMC8703868; doi:10.3390/molecules26247616)
Supplement: Supplementary file 1 [file molecules-26-07616-s001.zip › Table S1 supplementary material.pdf]

Table S1. Comparison of the significance of differences of the antioxidant potential (DPPH), Total Polyphenol Content (TPC), Total Flavonoid Content (TFC), pH and soluble solids of the analyzed samples during the fermentation process (after the 1st and 2nd month of fermentation).

**Grape variety**	**p- value**
After 1st month of fermentation (variant A- without added sugar)	
**DPPH**	
Solaris *vs.* Souvignier gris	* (0.000011)
Johanniter *vs.* Souvignier gris	* (0.000554)
**TPC**	
Solaris *vs.* Johanniter	* (0.015815)
Solaris *vs.* Souvignier gris	* (0.001463)
**pH**	
Solaris *vs.* Johanniter	* (0.009101)
Solaris *vs.* Souvignier gris	* (0.043916)
**Soluble solids**	
Solaris *vs.* Johanniter	* (0.000125)
Johanniter *vs.* Souvignier gris	* (0.000058)
After 1st month of fermentation (variant B- with added sugar)	
**(DPPH)**	
Solaris *vs.* Souvignier gris	* (0.000093)
Johanniter *vs.* Souvignier gris	* (0.0000101)
**TPC**	
Solaris *vs.* Souvignier gris	* (0.015815)
Johanniter *vs.* Souvignier gris	* (0.001463)
**TFC**	
Solaris *vs.* Johanniter	*(0.000375)

Solaris *vs.* Souvignier gris *(0.043916)

pH

Solaris *vs.* Johanniter *(0.012039)

Solaris *vs.* Souvignier gris *(0.026723)

Soluble solids

Solaris *vs.* Souvignier gris *(0.015815)

Johanniter *vs.* Souvignier gris *(0.015815)

<u>After 2nd month of fermentation (variant A- without added sugar)</u>

DPPH

Solaris *vs.* Johanniter *(0.048857)

Solaris *vs.* Souvignier gris *(0.000019)

TPC

Solaris *vs.* Johanniter *(0.029914)

Johanniter *vs.* Souvignier gris *(0.000008)

TFC

Solaris *vs.* Souvignier gris *(0.000810)

Johanniter *vs.* Souvignier gris *(0.002583)

pH

Solaris *vs.* Johanniter *(0.000086)

Johanniter *vs.* Souvignier gris *(0.000086)

Soluble solids

Solaris *vs.* Johanniter *(0.001463)

Johanniter *vs.* Souvignier gris *(0.002768)

<u>After 2nd month of fermentation (variant B- with added sugar)</u>

DPPH

Solaris *vs.* Souvignier gris *(0.000086)

Johanniter *vs.* Souvignier gris	*(0.000086)
**TPC**	
Solaris *vs.* Johanniter	*(0.010976)
Johanniter *vs.* Souvignier gris	*(0.031633)
**TFC**	
Solaris *vs.* Souvignier gris	*(0.014894)
Johanniter *vs.* Souvignier gris	*(0.008018)
**pH**	
Solaris *vs.* Johanniter	*(0.015815)
Solaris *vs.* Souvignier gris	*(0.015815)
**Soluble solids**	
Solaris *vs.* Souvignier gris	*(0.026723)
Johanniter *vs.* Souvignier gris	*(0.012039)

* statistically significant differences at $p \leq 0.05$;
