# Supplementary material for: The Influence of Time and Storage Conditions on the Antioxidant Potential and Total Phenolic Content in Homemade Grape Vinegars
Source: Molecules. 2021 Dec 15;26(24):7616. doi: 10.3390/molecules26247616 (PMC8703868; doi:10.3390/molecules26247616)
Supplement: Supplementary file 1 [file molecules-26-07616-s001.zip › Table S3 supplementary material.pdf]

Table S3. Comparison of the significance of the differences of the antioxidant potential (DPPH), Total Polyphenol Content (TPC), Total Flavonoid Content (TFC), pH and soluble solids of the analyzed samples during the storage process at room temperature.

**Grape variety**	**p- value**
Time point '0' (variant A- without added sugar)	
**DPPH**	
Solaris *vs.* Johanniter	* (0.048857)
Solaris *vs.* Souvignier gris	* (0.000019)
**TPC**	
Solaris *vs.* Johanniter	* (0.029914)
Solaris *vs.* Souvignier gris	* (0.000008)
**TFC**	
Solaris *vs.* Souvignier gris	* (0.029914)
Johanniter *vs.* Souvignier gris	* (0.002583)
**pH**	
Solaris *vs.* Johanniter	* (0.000086)
Johanniter *vs.* Souvignier gris	* (0.000086)
**Soluble solids**	
Solaris *vs.* Johanniter	* (0.001463)
Johanniter *vs.* Souvignier gris	* (0.002768)
After the 1st month of storage (variant A- without added sugar)	
**DPPH**	
Johanniter *vs.* Souvignier gris	* (0.000297)
**TPC**	
Solaris *vs.* Johanniter	* (0.000297)
**TFC**	
Johanniter *vs.* Souvignier gris	*(0.001076)

After the 3rd month of storage (variant A- without added sugar)

DPPH

Johanniter *vs.* Souvignier gris *(0.000572)

TPC

Solaris *vs.* Souvignier gris *(0.000297)

TFC

Solaris *vs.* Johanniter *(0.000297)

pH

Solaris *vs.* Souvignier gris *(0.005106)

Soluble solids

Solaris *vs.* Souvignier gris *(0.005106)

Time point '0' (variant B- with added sugar)

DPPH

Solaris *vs.* Souvignier gris *(0.000334)

Johanniter *vs.* Souvignier gris *(0.000164)

TPC

Solaris *vs.* Johanniter *(0.009176)

Solaris *vs.* Souvignier gris *(0.000001)

TFC

Solaris *vs.* Souvignier gris *(0.006390)

Johanniter *vs.* Souvignier gris *(0.009078)

pH

Solaris *vs.* Johanniter *(0.007334)

Solaris *vs.* Souvignier gris *(0.047902)

Johanniter *vs.* Souvignier gris *(0.000001)

Soluble solids

Solaris *vs.* Johanniter	*(0.000001)

After the 1st month of storage (variant B- with added sugar)

DPPH

Johanniter *vs.* Souvignier gris	*(0.001293)

TPC

Johanniter *vs.* Souvignier gris	*(0.000777)

TFC

Johanniter *vs.* Souvignier gris	*(0.000777)

Soluble solids

Solaris *vs.* Souvignier gris	*(0.032362)

After the 3rd month of storage (variant B- with added sugar)

DPPH

Solaris *vs.* Johanniter	*(0.000874)

TPC

Solaris *vs.* Johanniter	*(0.044883)
Johanniter *vs.* Souvignier gris	*(0.001973)

TFC

Solaris *vs.* Johanniter	*(0.000297)

pH

Solaris *vs.* Souvignier gris	*(0.005106)

Soluble solids

Solaris *vs.* Souvignier gris	*(0.005106)

* statistically significant differences at $p \leq 0.05$;
